# Transcriptional activation of *ompA* in *Neisseria gonorrhoeae* mediated by the XRE family member protein NceR

**DOI:** 10.1128/mbio.01244-23

**Published:** 2023-06-30

**Authors:** Concerta L. Holley, Vijaya Dhulipala, Stavaros A. Maurakis, Ashley Nicole Greenawalt, Timothy D. Read, Cynthia N. Cornelissen, William M. Shafer

**Affiliations:** 1 Department of Microbiology and Immunology, Emory University School of Medicine, Atlanta, Georgia, USA; 2 Institute for Biomedical Sciences, Georgia State University, Atlanta, Georgia, USA; 3 Department of Medicine (Division of Infectious Diseases), Emory University School of Medicine, Atlanta, Georgia, USA; 4 The Emory Antibiotic Resistance Center, Emory University School of Medicine, Atlanta, Georgia, USA; 5 Laboratories of Bacterial Pathogenesis, Veterans Affairs Medical Center, Decatur, Georgia, USA; Harvard Medical School, Boston, Massachusetts, USA

**Keywords:** *Neisseria gonorrhoeae*, *ompA*, regulation, NceR, iron

## Abstract

Increasing antibiotic resistance of *Neisseria gonorrhoeae*, the causative agent of gonorrhea, is a growing global concern that has renewed vaccine development efforts. The gonococcal OmpA protein was previously identified as a vaccine candidate due to its surface exposure, conservation, stable expression, and involvement in host–cell interactions. We previously demonstrated that the transcription of *ompA* can be activated by the MisR/MisS two-component system. Interestingly, earlier work suggested that the availability of free iron also influences *ompA* expression, which we confirmed in this study. In the present study, we found that iron regulation of *ompA* was independent of MisR and searched for additional regulators. A DNA pull-down assay with the *ompA* promoter from gonococcal lysates obtained from bacteria grown in the presence or absence of iron identified an XRE (Xenobiotic Response Element) family member protein encoded by *NGO1982*. We found that an *NGO1982* null mutant of *N. gonorrhoeae* strain FA19 displayed a reduced level of *ompA* expression compared to the wild-type (WT) parent strain. Given this regulation, and the capacity of this XRE-like protein to regulate a gene involved in peptidoglycan biosynthesis (*ltgA*), along with its presence in other *Neisseria* sp., we termed the *NGO1982-*encoded protein as NceR (*Neisseria* cell envelope regulator). Critically, results from DNA-binding studies indicated that NceR regulates *ompA* through a direct mechanism. Thus, *ompA* expression is subject to both iron-dependent (NceR) and -independent (MisR/MisS) pathways. Hence, levels of the vaccine antigen candidate OmpA in circulating gonococcal strains could be influenced by transcriptional regulatory systems and the availability of iron.

**IMPORTANCE:**

Herein, we report that the gene encoding a conserved gonococcal surface-exposed vaccine candidate (OmpA) is activated by a heretofore undescribed XRE family transcription factor, which we term NceR. We report that NceR regulation of *ompA* expression in *N. gonorrhoeae* is mediated by an iron-dependent mechanism, while the previously described MisR regulatory system is iron-independent. Our study highlights the importance of defining the complexity of coordinated genetic and physiologic systems that regulate genes encoding vaccine candidates to better understand their availability during infection.

## INTRODUCTION

The strict human pathogen *Neisseria gonorrhoeae* (Ng)*,* the etiological agent of the sexually transmitted infection (STI) gonorrhea, causes an estimated 87 million infections globally per year (1). Gonorrhea is the second most commonly reported infectious disease in the USA. Since the introduction of sulfonamides in the late 1930s followed by penicillin, Ng has displayed the ability to express clinical resistance to all introduced antibiotics (2, 3), and multi-drug resistant isolates have been reported globally in recent years (4 - 6). As such, Ng is considered an urgent public health threat pathogen by both the Centers for Disease Control and Prevention and the World Health Organization (2, 5, 6). The crisis of continued emergence of antibiotic resistance expressed by Ng strains coupled with reduced efforts in the antibiotic development field has renewed interest in developing a gonorrhea vaccine (7).

The ideal antigen for a gonococcal vaccine should be surface-exposed, highly conserved, and stably produced. One such protein, a 23-kDa outer membrane protein termed OmpA was previously identified as a potential vaccine candidate (7). The Ng OmpA protein is not subject to phase or antigenic variation, is conserved in all examined Ng strains, contributes to Ng interactions with host cells, and has been implicated as a virulence factor (8, 9). Notably, convalescent sera from patients recognized recombinant OmpA (9), and *ompA* was shown to be differentially expressed during symptomatic, natural cervical infection in women compared to Ng grown *in vitro* (10). Taken together, these studies support the potential of OmpA as an antigen that could be exploited for vaccine development.

An important consideration for any antigen that could be used in a gonorrhea vaccine is whether its presence in circulating strains might vary due to transcriptional regulatory systems that respond to environmental stresses. In this respect, we previously showed that the MisR/MisS two-component regulatory system can enhance *ompA* expression due to the binding of the response regulator MisR upstream of the *ompA* promoter. We hypothesized that additional regulatory systems are involved in modulating *ompA* expression in Ng since levels of its transcripts were found to be dependent on the availability of free iron in growth media (11). In the present study, we identified an iron-dependent, MisR-independent alternate pathway for the regulation of *ompA*. We identified a transcriptional activator of *ompA* encoded by the *NGO1982* gene. We designated this protein as NceR (*Neisseria* cell envelope regulator) and demonstrated that it can activate *ompA* in an iron-dependent manner independent of MisR.

## RESULTS AND DISCUSSION

### Ng *ompA* expression is activated in an iron-dependent manner that is independent of MisR

**Fig 1:**
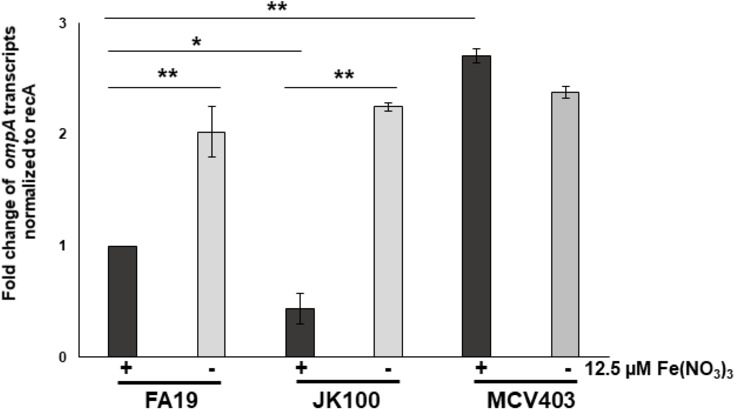
*ompA* is regulated in an iron-dependent, MisR-independent manner. qRT-PCR analysis of *ompA* transcripts in FA19, JK100, and MCV403 at the late-logarithmic phase of growth in the presence and absence of 12.5 µM Fe(NO_3_)_3_ in chemically defined medium (CDM). Error bars represent SDs from the means of three independent experiments. Normalized expression ratios were calculated using *recA* expression, and test values were further normalized against FA19 in the presence of Fe(NO_3_)_3_. The statistical significance of the results was determined by Student’s *t*-test and adjusted for multiple comparisons, *P < 0.05, **P < 0.001.

In our previous study, we determined that *ompA* is activated by the MisR/MisS two-component system with the response regulator MisR having the ability to bind to DNA sequences in the *ompA* promoter region (12). An examination of the gonococcal literature revealed that *ompA* was also differentially regulated when Ng is grown in iron-depleted media (13, 14). Interestingly, *ompA* expression was found to be enhanced in an Ng mutant lacking the iron-responsive transcriptional regulator Fur despite lacking a Fur-binding box (14). To determine if MisR was responsible for this observed increase in *ompA* expression in an iron-restricted manner, we examined *ompA* transcript and protein levels in WT strain FA19 and its isogenic *misR* null mutant (JK100) grown in the presence or absence of iron; the genotype of Ng strains used in this study are listed in Table S1. For this purpose, we utilized chemically defined media (CDM) to ensure the absence of iron (15); an iron-replete condition was created by the addition of 12.5 µM Fe(NO_3_)_3_ to the CDM. Quantitative reverse transcription PCR (qRT-PCR) analysis confirmed that *ompA* expression was elevated under iron-depleted conditions regardless of the presence of MisR (Fig. 1). Levels of OmpA, as well as iron-regulated TbpA outer membrane protein (Fig. S1), were elevated in the absence of iron further implicating an involvement of iron in modulating *ompA* gene expression (Fig. S1). As expected, *ompA* transcript and protein levels were increased in the Fur null mutant of FA19 and MCV403 (Fig. 1; Fig. S1, respectively). Thus, we concluded that an additional transcriptional factor(s) can control *ompA* expression in an iron-dependent manner.

### Identification of an XRE-family protein (NceR) capable of binding to the Ng *ompA* promoter region

To identify additional transcriptional factors capable of regulating *ompA* expression in an iron-dependent, MisR-independent manner, we utilized a DNA pull-down assay with a biotinylated *ompA* promoter DNA sequence-containing fragment followed by mass spectrometry (MS) to identify bound peptides of proteins having the ability to bind the target DNA (16). We compared lysates from isogenic strains FA19 and JK100 that were grown in the presence or absence of iron and focused on proteins that had increased peptide hits in the *misR* null mutant. A list of such MS peptide hits is shown in Table S2. In addition to MisR, we identified peptides within other known or presumed DNA-binding proteins including a protein encoded by the *ngo1982* gene. This gene would encode a heretofore uncharacterized protein in Ng that is a member of the Xenobiotic Response Element (XRE) family of transcriptional regulators. XRE family members are involved in a variety of metabolic and virulence functions (17, 18). Critically, the expression of *ngo1982* was shown to be repressed by Fur (14, 19) highlighting its potential for iron-dependent transcriptional regulation of *ompA*. Accordingly, we focused on the *ngo1982*-encoded protein as a candidate for regulating *ompA* expression by an iron-dependent mechanism.

**Fig 2:**
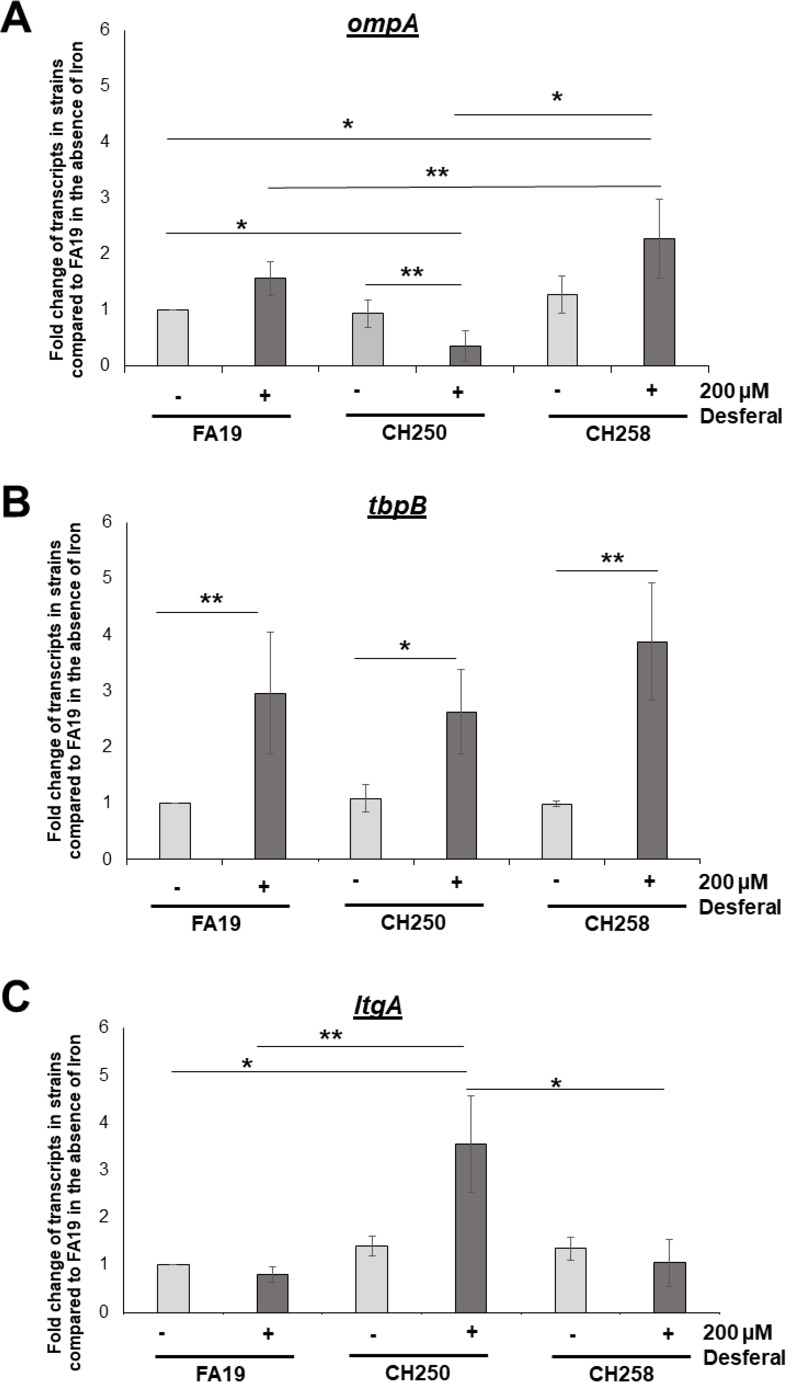
Deletion of *ngo1982* alters the expression of *ompA* (A) and *ltgA* (C) but not *tbpB* (B). qRT-PCR analysis of *ompA* transcripts in FA19, CH250, and complemented strain CH258 after 4 hours of incubation with 200 µM Desferal. Error bars represent SDs from the means of three independent experiments. Normalized expression ratios were calculated using *recA* expression, and test values were further normalized against FA19 in the absence of Desferal. The statistical significance of the results was determined by Student’s *t*-test and adjusted for multiple comparisons, *P < 0.05, **P < 0.001.

To test if the *ngo1982*-encoded, XRE-like protein could activate *ompA* expression in an iron-dependent manner, we constructed a deletion mutant in strain FA19 and compared *ompA* transcript levels in the deletion mutant (strain CH250) to that of the WT parent and an *ngo1982*-complemented strain (CH258). We found that in the presence of the iron chelator Desferal, loss of *ngo1982* significantly reduced *ompA* transcript levels (Fig. 2A). As expected, complementation of *ngo1982* (strain CH258) returned *ompA* transcript levels to that of WT strain FA19. Further, given that Fur represses *ngo1982* expression in the presence of iron (11), there was no difference in *ompA* transcript levels in the *ngo1982* null mutant strain (CH250) compared to the parental strain in the absence of Desferal (Fig. 2); hence, this observation likely explains why activation of *ompA* expression by the *ngo1982*-encoded regulatory protein is only observed under iron-depleted conditions.

During the course of our studies, we learned from J. Dillard (School of Medicine, University of Wisconsin, Madison, WI, personal communication) that the *ngo1982*-encoded protein also behaves as a repressor of *ltgA* expression in Ng; *ltgA* gene encodes the lytic transglycosylase A that processes peptidoglycan to produce extracellular toxic pro-inflammatory fragments (20, 21). In support of this, we found that in the presence of Desferal, loss of *ngo1982* gene significantly increased *ltgA* transcript levels (Fig. 2C) but had no impact on the expression of a control gene (*tbpB,* Fig. 2B), suggesting that it can also negatively control gene expression in Ng. Based on the ability of NGO1982 to regulate two genes involved in gonococcal cell envelope structure and that it is predicted (Fig. S3) to be encoded by other Neisserial genomes (including the human pathogen *N. meningitidis*), we (W. Shafer and J. Dillard) agreed to name the gene *nceR* (*N* eisseria cell envelope regulator). Bioinformatic analysis revealed that *nceR* is the last gene within a predicted six-gene operon (Fig. S4A). A putative promoter for the transcription of this operon, as identified in the MS11 transcriptome (22), was located 126 bp upstream of *ngo1987* (Fig. S4B).

### DNA-binding action of NceR

NceR is predicted to be a 99-amino acid protein with a calculated molecular weight of 11.43 kDa and an isoelectric point of 9.81 (Protein Reference ID: WP_010951376.1; https://www.uniprot.org/uniprotkb/Q5F5E7/entry). Bioinformatic analysis revealed that it is a member of the XRE family, one of the most abundant families of transcriptional regulation (23). The family has a common DNA-binding helix-turn-helix (HTH) motif that allows direct contact with the major groove of DNA (24, 25). The HTH motif in Ng shares similarity with family members from *Salmonella enterica*, *Klebsiella pneumoniae*, *Escherichia coli,* and *Streptococcus suis* (96%, 95%, 42%, and 35% amino acid sequence identity, respectively—see Fig. S5). In Ng, the HTH region spans amino acids 8–47 with the key DNA-binding sites for sequence-specific recognition predicted to be Q21, I22, S33, S36, T40, and G41 based on homology with crystallized DNA-binding proteins (26, 27). These amino acids are highly conserved across different *Neisseria* species including *N. meningitidis* and *N. lactamica* (Fig. S3). Phyre2 was used to predict the structure of NceR based on crystal structures of closely related proteins (Fig. S6) (28). CastP was used to predict the DNA-binding pocket which aligns with the HTH region further highlighting the importance of this region (29). Bioinformatic analysis of publicly available whole-genome sequences (30) from 767 clinical isolates revealed 17 protein allele groups in Ng including some variants with mutations at residues S36 and G41, which are positioned within the HTH domain (Table S3; Fig. S3). The most predominant allele group was WP_002215072.1 [AA mutations: M19L-L60I-A93S-G99S (15% overall)], but these residues are not located in the HTH domain. Overall, NceR, particularly in the HTH domain, is highly conserved across strains.

**Fig 3:**
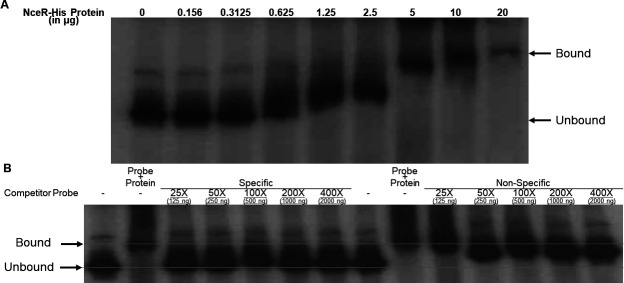
NceR binds to the *ompA* promoter region in a specific manner. (A) Shown is an EMSA demonstrating NceR binding to the *ompA* promoter. Lane 1, radiolabeled probe alone (5 ng); lanes 2–8, radiolabeled probe plus increasing concentrations of purified NceR from 0.156 to 20 µg. (B) Shown is a competitive EMSA demonstrating NceR-binding specificity to the *ompA* promoter. Lanes 1 and 8, radiolabeled probe alone (5 ng); lanes 2 and 9, radiolabeled probe plus NceR-His (5 µg); lanes 3–7, radiolabeled probe plus increasing concentrations of the unlabeled *ompA* probe (specific); lanes 10–14, radiolabeled probe plus increasing concentrations of the unlabeled *rnpB* probe (non-specific).

To determine if NceR directly regulates Ng *ompA* expression, as suggested by the DNA pull-down experiment, we performed DNA-binding studies using electrophoretic mobility shift assay (EMSA). We found that as little as 5 µg of recombinant His-tagged NceR was capable of binding to a 580-bp DNA fragment that is positioned upstream of the *ompA* translational start codon causing a complete shift in the target DNA (Fig. 3A). Moreover, in a competitive EMSA, NceR binding to the labeled target DNA was specific as it could only be inhibited by an unlabeled specific probe (Fig. 3B).

**Fig 4:**
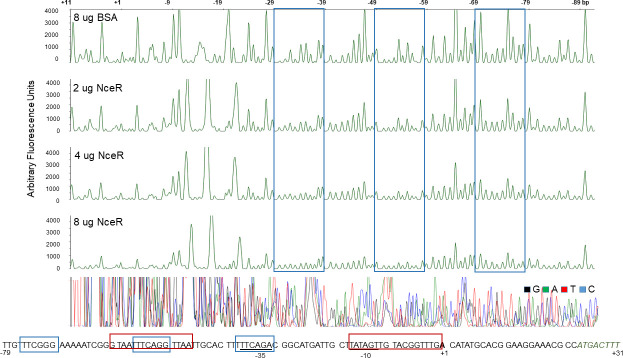
NceR protects three regions within and upstream of the *ompA* promoter. A DNA fragment spanning the *ompA* promoter region from nucleotide −89 to + 11 (relative to the TSS) was fluorescently labeled with 6-FAM (coding strand) and HEX (template strand) and incubated with BSA (control reaction) or NceR prior to digestion with DNAse I. The DNAse I digestion products were analyzed by capillary electrophoresis. The fluorescence signal corresponding to the HEX probe is shown on the *y*-axis of each electropherogram. Fragment coordinates (relative to the TSS) are shown on top. Electropherograms corresponding to 2, 4, and 8 µg of NceR and 8 µg BSA are shown. The NceR-protected region is boxed in blue. The MisR-binding sites are boxed in red. Sequencing reactions were manually generated (bottom panel). DNA sequence of the NceR-protected region is displayed below electropherograms.

**Fig 5:**
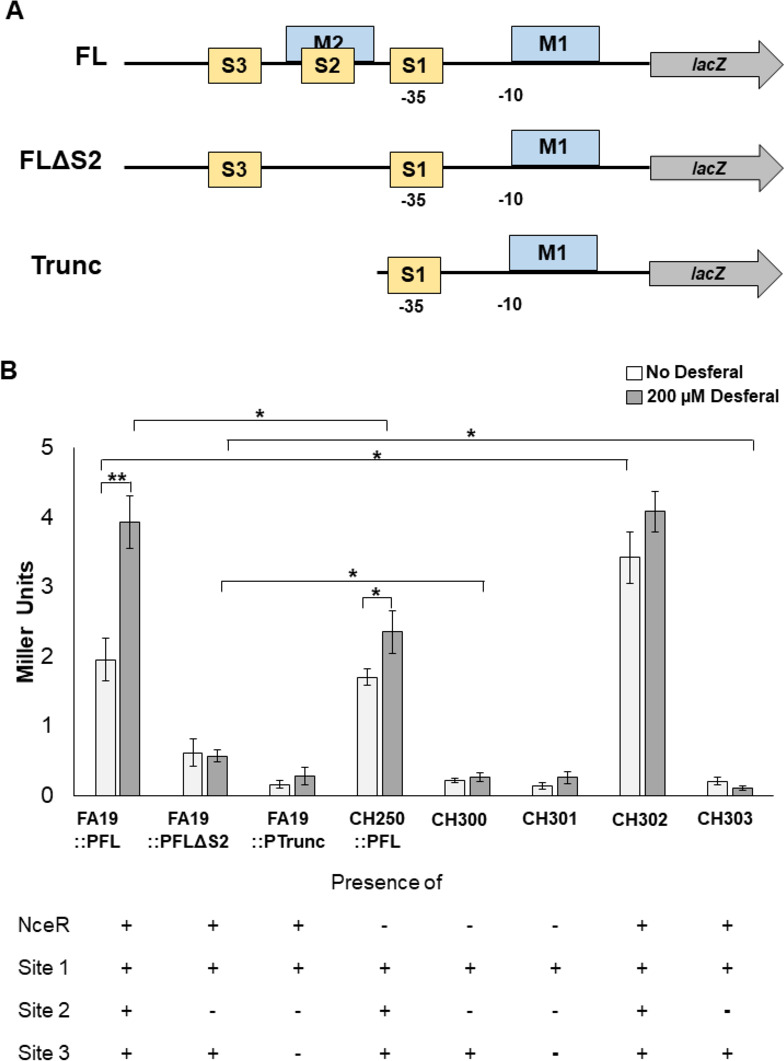
NceR primarily interacts with a single site within the *ompA* promoter. (A) The organization of the *ompA-*lacZ fusion constructs is depicted. The approximate locations of the predicted NceR-binding sites are indicated by boxes S1–S2–S3. The −10 and −35 hexamers are shown. Locations of the MisR-binding sites are indicated by M1–M2. (B) Effect of loss of NceR-binding sites on the expression of *ompA*. The specific β-galactosidase activity per milligram of total protein in cell extracts of reporter strains containing the *ompA-lacZ* fusions with disrupted NceR-binding site fusions (FLΔS2 and Trunc) in the FA19 and *nceR* null backgrounds. Results are the average of three independent experiments. Statistical significance was determined by analysis of variance; *P < 0.05, **P < 0.01.

Potential NceR-binding sites in the *ompA* promoter region were identified by DNAse I protection using a fluorescently labeled fragment of the *ompA* promoter region (positions −384 to +31 with respect to the *ompA* translational start site) and purified recombinant NceR-His protein. We found that NceR protected three sites in the targeted DNA sequence. One of these sites mapped within the −35 region of the *ompA* promoter, while the other two sites mapped upstream with one positioned within a previously described MisR-binding site (Fig. 4). To determine if all three NceR-binding sites are required for iron-mediated regulation of *ompA,* we took advantage of previously constructed *ompA* promoter-containing translational fusions that lack one or more of the distal binding sites. We compared activity in the absence of *nceR* at the *ompA* promoter with or without Desferal. Importantly, we found that there was a significant reduction in gene expression when the second site was deleted (Fig. 5). Moreover, loss of NceR (see strains CH300 and CH301) further reduced expression when the second site was deleted. Additionally, expression of NceR ectopically in strain CH302 abrogated iron-mediated repression of *ompA*. Collectively, the data suggest that binding site 2 is critical for NceR recognition and gene activation.

Given that OmpA is a gonococcal vaccine candidate, defining the mechanisms that control *ompA* expression should give insights as to its level on the Ng surface during infection. Our previous work showed that the regulation of *ompA* is dependent on activation by the MisR/MisS two-component system (12) with phosphorylated MisR binding near and within the *ompA* promoter. In this study, we identified a second pathway of *ompA* regulation in which transcription can be through a MisR/MisS- independent pathway, but in an iron-dependent manner. With the use of a DNA pull-down assay, we identified NceR, a member of the XRE family of bacterial transcriptional regulators, as an activator of *ompA* expression. We defined NceR’s interaction with the *ompA* promoter region, demonstrating that it has the capacity to bind primarily to a single site. Finally, we showed the impact of *ngo1982* deletion on the expression of *ompA* in iron-limited conditions.

XRE transcriptional regulators are a large family of proteins possessing HTH DNA-binding motifs (23) and are the second most commonly occurring regulator family in bacteria (31). Members of this family regulate genes involved in virulence, stress response, and metabolism, often galvanized by environmental cues (17, 18, 32 - 35). A previously studied XRE family member in Ng, the NHTF (*Neisseria* hypothetical transcription factor) protein, represses the expression of its downstream genes including a conserved nitrogen-response protein (32). In this way, NHTF, like other XRE members responds to environmental cues. Given XRE regulators and their roles in response to environmental changes, NceR’s potential function as an iron-responsive cell envelope regulator is consistent with XRE-mediated regulation of physiologic systems.

While NceR activation of *ompA* is iron-dependent, we do not yet fully understand the overall mechanism by which this regulatory pathway operates. However, we hypothesize that Fur regulates *ompA* indirectly. Fur has been shown to control several regulatory proteins including ArsR, MpeR, and OxyR (19) whose targets include genes involved in antimicrobial resistance, intracellular survival, and resistance to oxidative stress. When iron becomes limited, Fur becomes inactive and de-repression of targets can occur, freeing transcription of repressed genes. In iron-replete conditions, NceR is repressed by Fur (13) and remains inactive and unavailable to activate *ompA* until de-repression occurs. Therefore, Fur could indirectly regulate *ompA* adding *ompA* to the list of iron-responsive genes (14, 36). Further characterization of NceR, including determining its regulon and its interaction and overlap with the Fur regulon, would expand our understanding not only of NceR’s role in pathogenesis but also of XRE family proteins in *Neisseria* species overall.

The presence of OmpA on the Ng surface confers an advantage during infection in a female mouse model of infection and OmpA is involved in adherence and invasion into human epithelial cells which is critical for establishing an infection (8). Similarly, *Acinetobacter baumannii* strains expressing high levels of OmpA were significantly more invasive than those with lower levels of OmpA (37). Unlike in Ng, however, in *A. baumannii* OmpA is increased in iron-rich conditions (37) which may be because it serves as a porin that allows small molecules to pass through unperturbed (38, 39). It has been suggested that the *Acinetobacter* OmpA porin plays a role in cell homeostasis and cellular function under specific stress conditions (40). The Ng OmpA protein may play a similar role in the gonococcus that could explain why transcription of *ompA* can be activated by both MisR/MisS and NceR. NceR and MisR/MisS may therefore represent additional novel targets for therapeutic or preventative strategies to combat the ongoing global problem of Ng antibiotic resistance. In this regard, chemotherapeutic interventions that increase the activity of MisR/MisS or NceR may increase surface expression or OmpA resulting in increased immune visibility. In contrast, it is also possible that gonococci could develop *cis*-acting mutations in the operator sites used for MisR and/or NceR binding or *trans*-acting mutations in these regulators that dampen levels of *ompA* expression thereby resulting in reduced immune visibility.

**Fig 6:**
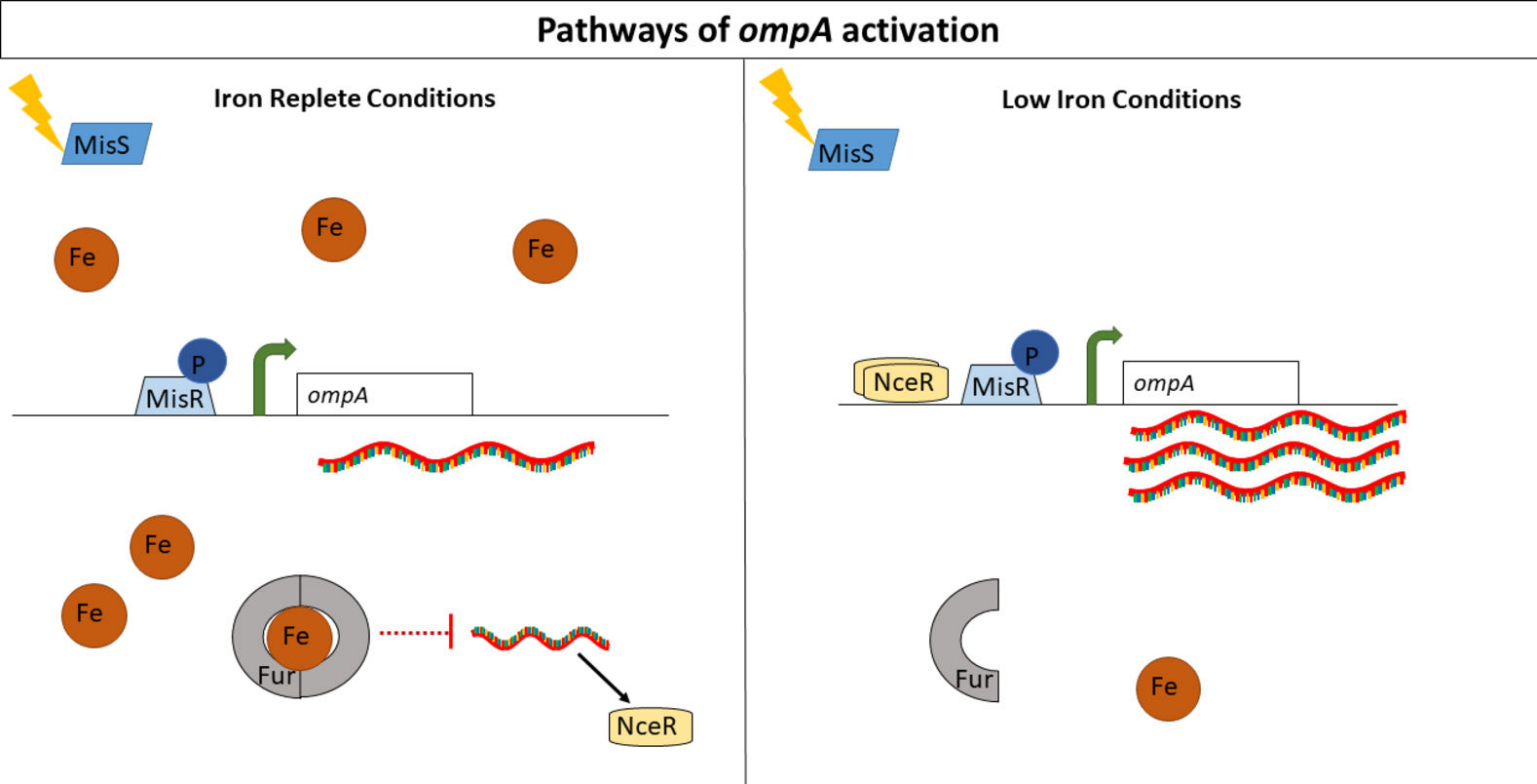
A proposed model for dual pathways of activation for the *ompA* gene. In the first pathway, a yet-unknown signal triggers the sensor kinase MisS to phosphorylate its response regulator MisR. MisR binds to the *ompA* promoter helping to recruit RNA polymerase and drive the transcription of *ompA.* MisR activation of the *ompA* promoter is independent of iron availability. Activation by NceR is iron-dependent. Under iron-replete conditions, Fur-Fe^2+^ inhibits the expression of NceR. When iron is low, Fur is no longer capable of repressing NceR expression, releasing NceR to bind and activate the *ompA* promoter. When iron is low, both MisR and NceR can interact with the *ompA* promoter, thus increasing *ompA* transcripts in iron-depleted conditions.

In conclusion, the data presented in this study suggest that Ng adjusts *ompA* expression depending on both the activation state of the MisR/MisS system (12) and environmental cues such as iron levels in the context of NceR; a model for these regulatory systems is summarized in Fig. 6. We suggest that these regulatory pathways may allow Ng to rapidly adapt to different *in vivo* signals and optimize protein expression patterns to enhance bacterial survival. Taken together, our study offers new insight into understanding the mechanisms Ng employs in the regulation of genes encoding vaccine candidates.

## MATERIALS AND METHODS

### Bacterial strains, plasmids, and primers

Ng strain FA19 and its isogenic genetic derivative strains, along with the plasmids used and their *E. coli* hosts, are listed in Table S1. The oligonucleotide primers used in this study are listed in Table S4. *E. coli* strains were routinely cultured on Luria-Bertani (LB) agar or in LB broth (Difco, Sparks, MD, USA) containing 50 µg/mL Kan, 100 µg/mL ampicillin, or 100 µg/mL chloramphenicol as necessary. Gonococci were grown on gonococcal base (GCB) agar (Difco, Sparks, MD) containing Kellogg’s supplements I and II at 37°C under 5.0% (vol/vol) CO_2_ (41). Liquid cultures of gonococci for growth assays were begun by inoculating plate-grown cells in pre-warmed GCB broth containing Kellogg’s supplements I and II and 0.043% (wt/vol) sodium bicarbonate and grown in a 37°C water bath with shaking. For iron-depleted growth, liquid cultures were grown in either GCB broth containing media containing Kellogg’s ssupplement I, 0.043% (wt/vol) sodium bicarbonate, and 200 μM Desferal or Chelex-treated CDM as described elsewhere (15).

### DNA pull-down and mass spectrometry

To prepare lysates for pull-down, FA19 and JK100 were grown in GC broth in the presence and absence of 200 µM Desferal for 3 hours. Cells were then harvested, resuspended in lysis buffer [20 mM HEPES pH 7.6, 1 mM EDTA, 10 mM (NH_4_)_2_SO_4_, 1 mM dithiothreitol (DTT), 0.1% (vol/vol) Tween 20, 30 mM KCl, 75 mM NaCl plus Roche’s cOmplete protease inhibitor cocktail], and lysed on a French press. A DNA fragment encoding the *ompA* promoter was amplified with the biotinylated primer ompA_PD_F and ompA_PD_R. The PCR product was purified using the QIAquick PCR purification kit (QIAGEN) and confirmed by DNA sequencing. A mix containing 100 µg of streptavidin-coupled dynabeads and 2.5 µg of biotinylated DNA was incubated for 90 minutes at room temperature. The dynabeads-DNA complex was washed to remove unbound DNA and resuspended in 500 µL of bacterial lysate containing 1.5 mg of total proteins. The mixture was incubated for 90 minutes at room temperature, and the dynabeads-DNA-protein complexes were captured and washed five times with wash buffer (10 mM Tris-HCl pH 7.5, 1 mM EDTA, 0.1 M NaCl). Pulled down proteins were eluted by resuspension and boiling for 5 minutes in 40 µL of Laemmli sample buffer. Samples were loaded and run 1 cm into Any kD Mini-PROTEAN TGX precast gels (Bio-Rad) and stained with Coomassie brilliant blue. The protein band was excised from the gel and submitted for liquid chromatography with tandem mass spectrometry (LC-MS/MS) analysis. Proteins were identified by peptide mapping to an FA19 proteomic database using Thermo Scientific Protein Discover 1.4 software.

### Construction of *ngo1982* mutants

An unmarked Δ*ngo1982* mutant was constructed as follows. Primer pairs 1982start/1982F1 and 1982stop/1982R1 were used to amplify the upstream and downstream sequences of the *ngo1982* gene. The two PCR products were ligated together at the XbaI site and cloned into pBad TOPO-TA. The confirmed plasmid was designated pCH31. pUNCH937 was digested with HindIII and XbaI to extract the streptomycin sensitivity conferring *rpls/Cat* cassette which was inserted into vector pCH31 to create pCH32. pCH32 was transformed into the streptomycin-resistant strain FA19 *rpsl.* Streptomycin-sensitive, chloramphenicol-resistant colonies were isolated. To create the unmarked deletion mutant, the streptomycin sensitive strains were then transformed with pCH31 to remove the sensitive allele and chloramphenicol resistance cassette. Deletion of the gene and loss of the cassette was confirmed by PCR and sequencing. This final strain was designated CH250.

### Complementation of the Δ*ngo1982* mutant

Strain CH250 was complemented as previously described using the pGCC4 complementation vector. The entire *ngo1982* gene and flanking regions were amplified from FA19 genomic DNA using primers 1982pacI and 1982pmeI and inserted into digested pGCC4 vector (42). The resulting plasmid was used to transform CH250, and colonies were selected on chloramphenicol 1 µg/mL and verified by PCR and sequencing. Transformants were verified by PCR and sequencing using primers lctp and aspC1.

### Analysis of Ng transcripts

For the measurement of target gene expression, gonococci were harvested at late-log phase and the pellets were stored at −70°C. RNA was purified by Trizol extraction as per manufacturer instructions (Thermo Fisher Scientific, Waltham, MA, USA) followed by Turbo DNA-free (Ambion, Austin, TX, USA) treatment. cDNA was generated using a QuantiTect reverse transcriptase kit (Qiagen, Venlo, The Netherlands). We validated our qRT-PCR methods by examining primer efficiency, primer specificity (melt temperature), and linear dynamic range for each primer pair utilized herein. For qRT-PCR analysis, the normalized expression of each target gene was calculated using *recA* as a housekeeping reference gene (43). All qRT-PCRs were performed in technical duplicates and biological quadruplicates.

### Purification of recombinant NceR-His protein

The coding sequence of *ngo1982* was amplified with primers 1982HisF and 1982HisR. The PCR product was digested with BamHI and NdeI and then cloned into pET-15b which had been digested with the same enzymes to yield pCH30. The plasmid was purified and transformed into *E. coli* expression strain BL21(DE3). A NceR-His fusion protein was purified as per the manufacturer’s protocol using a (Ni+2-NTA) column (Millipore Sigma, Burlington, MA, USA). Protein was eluted in buffer containing 200 mM imidazole, dialyzed to remove imidazole using 10 mM phosphate-buffered saline (137 mM NaCl, 2.7 mM KCl, 10 mM Na_2_HPO_4_, 2 mM KH_2_PO_4_), and concentrated. DTT and glycerol were added to a final concentration of 1 mM and 10% (vol/vol), respectively. The purity of recombinant NceR was confirmed by SDS‐PAGE electrophoresis staining with Coomassie blue.

### Western blotting

Detection of TbpA and OmpA was performed as follows. Briefly, whole cell lysates were prepared in 2× SDS loading dye [100 mM Tris-HCl, pH 6.8, 4% (wt/vol) SDS, 0.2% (wt/vol) bromophenol blue, 20% glycerol, 200 mM DTT]. Proteins were separated on a mini-protean TGX stain-free gel (Bio-Rad, Hercules, CA, USA) and transferred to nitrocellulose or Immobilon membrane (Millipore, Burlington, MA, USA) and transferred for 16 hours at 28 mA using wet transfer. A Ponceau stain was conducted before blocking in 5% (wt/vol) milk low-salt TBS buffer [50 mM Tris, 0.15 M NaCl, 0.05% (vol/vol) Tween 20, pH 7.4]. Blots were probed with either polyclonal antibody to TbpA (1:1,000) or polyclonal antibody to OmpA (1:1,000) (17). Blots were then washed with TBS buffer before incubation with secondary anti-rabbit antibody (1:5,000) dilution. Blots were visualized with Dura-ECL reagents (Thermo Fisher Scientific, Waltham, MA, USA) or Immun-Blot Opti-4CN colorimetric kit (Bio-Rad, Hercules, CA, USA).

### EMSA for the detection of NceR binding to target DNA

A DNA probe containing the *ompA* promoter region was amplified by PCR from FA19 genomic DNA using the primers pOmpA2F and pOmpAR. For radiolabeled probes, the indicated PCR product was labeled with (γ32P)-dATP using T4 polynucleotide kinase (New England Biolabs, Ipswitch, MA, USA). Five nanograms of the labeled DNA fragment was incubated with increasing concentrations of purified NceR-His. For competition assays, the unlabeled *ompA* probe or an unlabeled PCR product using RnpB1F and RnpB1R primers (non-specific *rnpB* probe) were incubated with protein for 15 minutes before the addition of the radiolabeled probe. All samples were subjected to electrophoresis in a 6% native PAGE at 4°C, pH 8.5 followed by autoradiography.

### DNAse I protection assays

A PCR fragment spanning the *ompA* promoter was amplified using the 6-carboxyfluorescein (FAM)- and 6-carboxy-2′,4,4′,5′,7,7′-hexachlorofluorescein (HEX)-labeled primers [FAM]ompAFL and [HEX]ompR. NceR protein binding to the labeled DNA probe and DNAse I digestion reactions were performed as described previously (44). Detection of the DNAse I digestion peaks was carried out in a 3730 capillary sequencer (Applied Biosystems, Waltham, MA, USA) by Azenta Life Sciences (Chelmsford, MA, USA), and the alignment of the corresponding electropherograms was generated using GeneMapper Software Version 4.0 (Applied Biosystems). Negative control reactions were done using bovine serum albumin (BSA) at the same mass concentration used for NceR. Phosphorylated MisR protein was prepared as previously described as a positive control (45). A DNA template was amplified with primers ompAFL and ompR to generate a sequence ladder for each strand as previously described (46). The final electropherograms of the sequencing reactions were horizontally aligned with those generated in the footprint using GeneMapper Software Version 4.0 (Applied Biosystems). As confirmation, the HEX/FAM-labeled DNA probe was subjected to Sanger sequencing using primer pompR.

### Bioinformatic analysis of NceR proteins in a database of sequenced clinical genomes

We used the ncbi-genome-download tool (version 0.3.0; https://github.com/kblin/ncbi-genome-download) to download a database of proteins from *N. gonorrhoeae* genomes using the following command: ncbi- genome-download –species-taxids 485 –formats ‘protein-fasta’ bacteria. The proteomes of 783 genomes were downloaded. We searched for orthologs of NceR (WP_010951376.1) against this database of proteins from 783 public genome projects downloaded using BLASTP (version 2.12) and selected those with a threshold of at least 95% sequence identity. Seven hundred sixty-seven genomes had an NceR ortholog, and these were represented by 17 individual NCBI protein IDs, equivalent to amino acid sequence alleles.

### β-galactosidase assays

A plasmid containing the full-length *ompA* promoter translationally fused to the truncated, promoter-less *lacZ* gene in pLES94 was constructed previously (12). The plasmid was used to transform the WT strain FA19 and its mutants to generate strains FA 19::P*FL*, CH250::P*FL*, and CH258::P*FL.* Gonococcal transformants were selected on GCB agar containing 1 µg/mL of chloramphenicol and further verified by PCR. We utilized previously constructed plasmids in which either the S2 site (pCH25) or both the S2 and S1 site were deleted (pCH23) to determine the primary binding site for NceR. These plasmids were transformed into CH250 and CH258 to create strains CH300, CH301, CH302, and CH303. All strains were confirmed by sequencing. Strains containing *lacZ* translational fusions were grown overnight on GCB agar plates containing 1 µg/mL of chloramphenicol. For assays, cells were grown in GC broth in 96-well plates (Costar No. 3370; Corning, Durham, NC, USA) with Kellogg’s Supplement 1, sodium bicarbonate, 1 µg/mL of chloramphenicol, and 10 mM MgCl_2_. Half of the wells received 100 µM Desferal and half received 12.5 mM Fe(NO_3_)_3_. After 6 hours of incubation at 37°C, the OD600 was measured using a microplate reader. The plate was then stored in the 4°C on ice overnight. For processing, the cell culture was resuspended by pipetting up and down 10 times and 18.75 µL was transferred to a new microplate (Costar No. 9017, Corning) containing 56.25 µL CelLytic B Cell lysis reagent (Sigma, St. Louis, MO, USA). The plate was incubated for 30 minutes at 26°C and then 62.5 µL B-gal buffer (60 mM Na_2_HPO_4_, 40 mM NaH_2_PO_4_ pH 7.0, 10 mM KCl, 1 mM MgSO_4_) and 37.5 µL ortho-nitrophenyl-β-galactoside was added. The plate was then incubated until yellow color developed and the reaction stopped with 73 µL of 1 M Na_2_CO_3_. The OD420 was read on the plate reader. β-galactosidase activities are given in Miller units using the formula [1,000 × OD_420 nm_/(*t* × *v* × OD_600 nm_)], where *t* is the reaction time in minutes and *v* is the volume of cell lysates in milliliter per reaction.

### Statistical methods

All the data were expressed as means with standard deviations. Statistical significance between all quantitative data was analyzed by Student *t*-tests or one-way analysis of variance followed by Tukey’s honestly significant difference *post hoc* test. Statistical significance was set at *P* < 0.05.

## Data Availability

The data sets that supported the findings of this study are available from the corresponding author upon request.

## SUPPLEMENTAL MATERIAL

Supplemental material mbio.01244-23-s0001.pdf Fig. S1 to S6 and Tables S1 to S4.

ASM does not own the copyrights to Supplemental Material that may be linked to, or accessed through, an article. The authors have granted ASM a non-exclusive, world-wide license to publish the Supplemental Material files. Please contact the corresponding author directly for reuse.

## References

[1] Centers for Disease Control and Prevention . 2019. Sexually transmitted disease surveillance 2018. U.S. Department of Health and Human Services, Atlanta. doi:10.15620/cdc.79370

[2] Centers for Disease Control and Prevention . 2019. Sexually transmitted disease surveillance 2019: Gonococcal isolate surveillance project (GISP). U.S. Department of Health and Human Services. Available from: https://www.cdc.gov/std/statistics/gisp-profiles/default.htm

[3] Centers for Disease Control and Prevention . 2021. Sexually transmitted disease surveillance 2019: Gonococcal isolate surveillance project (GISP) supplement and profiles. U.S. Department of Health and Human Services, Atlanta.

[4] Tien V , Punjabi C , Holubar MK . 2020. Antimicrobial resistance in sexually transmitted infections. J Travel Med 27:taz101. doi:10.1093/jtm/taz101 31840758

[5] Unemo M , Lahra MM , Escher M , Eremin S , Cole MJ , Galarza P , Ndowa F , Martin I , Dillon J-A , Galas M , Ramon-Pardo P , Weinstock H , Wi T . 2021. WHO global antimicrobial resistance surveillance (GASP/GLASS) for Neisseria gonorrhoeae 2017-2018: a retrospective observational study. Lancet Microbe 2:e627–e636. doi:10.1016/S2666-5247(21)00171-3 35544082

[6] Unemo M , Lahra MM , Cole M , Galarza P , Ndowa F , Martin I , Dillon J-A , Ramon-Pardo P , Bolan G , Wi T . 2019. World Health Organization global gonococcal antimicrobial surveillance program (WHO GASP): review of new data and evidence to inform international collaborative actions and research efforts. Sex Health 16:412–425. doi:10.1071/SH19023 31437420 PMC7035961

[7] Rice PA , Shafer WM , Ram S , Jerse AE . 2017. Neisseria gonorrhoeae: drug resistance, mouse models, and vaccine development. Annu Rev Microbiol 71:665–686. doi:10.1146/annurev-micro-090816-093530 28886683

[8] Serino L , Nesta B , Leuzzi R , Fontana MR , Monaci E , Mocca BT , Cartocci E , Masignani V , Jerse AE , Rappuoli R , Pizza M . 2007. Identification of a new OmpA-like protein in Neisseria gonorrhoeae involved in the binding to human epithelial cells and in vivo colonization. Mol Microbiol 64:1391–1403. doi:10.1111/j.1365-2958.2007.05745.x 17542928

[9] Starnino S , Leuzzi R , Ghisetti V , De Francesco MA , Cusini M , Impara G , Galluppi E , Pizza M , Stefanelli P . 2010. Molecular analysis of two novel Neisseria gonorrhoeae virulent components: the macrophage infectivity potentiator and the outer membrane protein A. New Microbiol 33:167–170. 20518279

[10] McClure R , Nudel K , Massari P , Tjaden B , Su X , Rice PA , Genco CA . 2015. The gonococcal transcriptome during infection of the lower genital tract in women. PLoS One 10:e0133982. doi:10.1371/journal.pone.0133982 26244506 PMC4526530

[11] Ducey TF , Jackson L , Orvis J , Dyer DW . 2009. Transcript analysis of nrrF, a Fur repressed sRNA of Neisseria gonorrhoeae. Microb Pathog 46:166–170. doi:10.1016/j.micpath.2008.12.003 19162160 PMC4890603

[12] Holley CL , Ayala JC , Shafer WM . 2020. Transcriptional control of the gonococcal ompA gene by the MisR/MisS two-component regulatory system. Sci Rep 10:9425. doi:10.1038/s41598-020-66382-2 32523077 PMC7286886

[13] Ducey TF , Carson MB , Orvis J , Stintzi AP , Dyer DW . 2005. Identification of the iron-responsive genes of Neisseria gonorrhoeae by microarray analysis in defined medium. J Bacteriol 187:4865–4874. doi:10.1128/JB.187.14.4865-4874.2005 15995201 PMC1169496

[14] Jackson LA , Ducey TF , Day MW , Zaitshik JB , Orvis J , Dyer DW . 2010. Transcriptional and functional analysis of the Neisseria gonorrhoeae Fur regulon. J Bacteriol 192:77–85. doi:10.1128/JB.00741-09 19854902 PMC2798271

[15] Maurakis S , Cornelissen CN . 2020. Metal-limited growth of Neisseria Gonorrhoeae for characterization of metal-responsive genes and metal acquisition from host ligands. J Vis Exp 157:e60903. doi:10.3791/60903 PMC7386066 32202529

[16] Ayala JC , Wang H , Benitez JA , Silva AJ . 2018. Molecular basis for the differential expression of the global regulator VieA in Vibrio cholerae biotypes directed by H-NS, LeuO and quorum sensing. Mol Microbiol 107:330–343. doi:10.1111/mmi.13884 29152799 PMC5777889

[17] Liu G , Gao T , Zhong X , Ma J , Zhang Y , Zhang S , Wu Z , Pan Z , Zhu Y , Yao H , Liu Y , Lu C , Freitag NE . 2020. The novel streptococcal transcriptional regulator XtgS negatively regulates bacterial virulence and directly represses PseP transcription. Infect Immun 88:e00035-20. doi:10.1128/IAI.00035-20 32690636 PMC7504938

[18] Trouillon J , Ragno M , Simon V , Attrée I , Elsen S , van Wezel GP . 2021. Transcription inhibitors with XRE DNA-binding and cupin signal-sensing domains drive metabolic diversification in Pseudomonas. mSystems 6:e00753-20. doi:10.1128/mSystems.00753-20 33436508 PMC7901475

[19] Yu C , McClure R , Nudel K , Daou N , Genco CA . 2016. Characterization of the Neisseria gonorrhoeae iron and Fur regulatory network. J Bacteriol 198:2180–2191. doi:10.1128/JB.00166-16 27246574 PMC4966432

[20] Schaub RE , Chan YA , Lee M , Hesek D , Mobashery S , Dillard JP . 2016. Lytic transglycosylases LtgA and LtgD perform distinct roles in remodeling, recycling and releasing peptidoglycan in Neisseria gonorrhoeae. Mol Microbiol 102:865–881. doi:10.1111/mmi.13496 27608412 PMC5463997

[21] Cloud-Hansen KA , Hackett KT , Garcia DL , Dillard JP . 2008. Neisseria gonorrhoeae uses two lytic transglycosylases to produce cytotoxic peptidoglycan monomers. J Bacteriol 190:5989–5994. doi:10.1128/JB.00506-08 18567658 PMC2519546

[22] Remmele CW , Xian Y , Albrecht M , Faulstich M , Fraunholz M , Heinrichs E , Dittrich MT , Müller T , Reinhardt R , Rudel T . 2014. Transcriptional landscape and essential genes of Neisseria gonorrhoeae. Nucleic Acids Res 42:10579–10595. doi:10.1093/nar/gku762 25143534 PMC4176332

[23] Wintjens R , Rooman M . 1996. Structural classification of HTH DNA-binding domains and protein-DNA interaction modes. J Mol Biol 262:294–313. doi:10.1006/jmbi.1996.0514 8831795

[24] Schumacher MA , Piro KM , Xu W , Hansen S , Lewis K , Brennan RG . 2009. Molecular mechanisms of HipA-mediated multidrug tolerance and its neutralization by HipB. Science 323:396–401. doi:10.1126/science.1163806 19150849 PMC2764309

[25] Watkins D , Hsiao C , Woods KK , Koudelka GB , Williams LD . 2008. P22 c2 repressor-operator complex: mechanisms of direct and indirect readout. Biochemistry 47:2325–2338. doi:10.1021/bi701826f 18237194

[26] Lu S , Wang J , Chitsaz F , Derbyshire MK , Geer RC , Gonzales NR , Gwadz M , Hurwitz DI , Marchler GH , Song JS , Thanki N , Yamashita RA , Yang M , Zhang D , Zheng C , Lanczycki CJ , Marchler-Bauer A . 2020. CDD/SPARCLE: the conserved domain database in 2020. Nucleic Acids Res 48:D265–D268. doi:10.1093/nar/gkz991 31777944 PMC6943070

[27] Luscombe NM , Austin SE , Berman HM , Thornton JM . 2000. An overview of the structures of protein-DNA complexes. Genome Biol 1:REVIEWS001. doi:10.1186/gb-2000-1-1-reviews001 11104519 PMC138832

[28] Kelley LA , Mezulis S , Yates CM , Wass MN , Sternberg MJE . 2015. The Phyre2 web portal for protein modeling, prediction and analysis. Nat Protoc 10:845–858. doi:10.1038/nprot.2015.053 25950237 PMC5298202

[29] Tian W , Chen C , Lei X , Zhao J , Liang J . 2018. CASTp 3.0: computed atlas of surface topography of proteins. Nucleic Acids Res 46:W363–W367. doi:10.1093/nar/gky473 29860391 PMC6031066

[30] Bethesda (MD): National Library of Medicine (US)NCfBI . 2004. Whole genome database [Internet]. Bethesda (MD): National Library of Medicine (US), National Center for Biotechnology Information. Available from: https://www.ncbi.nlm.nih.gov/genome/ ed. Retrieved 31 Mar 2023.

[31] Santos CL , Tavares F , Thioulouse J , Normand P . 2009. A phylogenomic analysis of bacterial helix-turn-helix transcription factors. FEMS Microbiol Rev 33:411–429. doi:10.1111/j.1574-6976.2008.00154.x 19076237

[32] Wang H-C , Ko T-P , Wu M-L , Ku S-C , Wu H-J , Wang AH-J . 2012. Neisseria conserved protein DMP19 is a DNA mimic protein that prevents DNA binding to a hypothetical nitrogen-response transcription factor. Nucleic Acids Res 40:5718–5730. doi:10.1093/nar/gks177 22373915 PMC3384305

[33] Zhang Y , Liang S , Pan Z , Yu Y , Yao H , Liu Y , Liu G . 2022. XRE family transcriptional regulator XtrSs modulates Streptococcus suis fitness under hydrogen peroxide stress. Arch Microbiol 204:244. doi:10.1007/s00203-022-02854-5 35386008

[34] Lu H , Wang L , Li S , Pan C , Cheng K , Luo Y , Xu H , Tian B , Zhao Y , Hua Y . 2019. Structure and DNA damage-dependent derepression mechanism for the XRE family member DG-DdrO. Nucleic Acids Res 47:9925–9933. doi:10.1093/nar/gkz720 31410466 PMC6765133

[35] Ren X , Chen Z , Niu P , Han W , Ding C , Yu S . 2021. XRE-type regulator BioX acts as a negative transcriptional factor of biotin metabolism in riemerella anatipestifer. J Bacteriol 203:e0018121. doi:10.1128/JB.00181-21 33972354 PMC8407344

[36] Vélez Acevedo RN , Ronpirin C , Kandler JL , Shafer WM , Cornelissen CN . 2014. Identification of regulatory elements that control expression of the tbpBA operon in Neisseria gonorrhoeae. J Bacteriol 196:2762–2774. doi:10.1128/JB.01693-14 24837286 PMC4135677

[37] Liu H , Cao CY , Qiu FL , Huang HN , Xie H , Dong R , Shi YZ , Hu XN . 2021. Iron-rich conditions induce OmpA and virulence changes of Acinetobacter baumannii. Front Microbiol 12:725194. doi:10.3389/fmicb.2021.725194 34675899 PMC8525545

[38] Iyer R , Moussa SH , Durand-Réville TF , Tommasi R , Miller A . 2018. Acinetobacter baumannii OmpA is a selective antibiotic permeant porin. ACS Infect Dis 4:373–381. doi:10.1021/acsinfecdis.7b00168 29260856

[39] Sugawara E , Nikaido H . 2012. OmpA is the principal nonspecific slow porin of Acinetobacter baumannii. J Bacteriol 194:4089–4096. doi:10.1128/JB.00435-12 22636785 PMC3416538

[40] Shahryari S , Talaee M , Haghbeen K , Adrian L , Vali H , Shahbani Zahiri H , Noghabi KA . 2021. New provisional function of OmpA from Acinetobacter sp. strain SA01 based on environmental challenges. mSystems 6:e01175-20. doi:10.1128/mSystems.01175-20 33436517 PMC7901484

[41] Dillard JP . 2011. Genetic manipulation of Neisseria gonorrhoeae. Curr Protoc Microbiol Chapter 4:Unit4A.2. doi:10.1002/9780471729259.mc04a02s23 PMC4549065 22045584

[42] Skaar EP , Lazio MP , Seifert HS . 2002. Roles of the recJ and recN genes in homologous recombination and DNA repair pathways of Neisseria gonorrhoeae. J Bacteriol 184:919–927. doi:10.1128/jb.184.4.919-927.2002 11807051 PMC134828

[43] Pfaffl MW . 2001. A new mathematical model for relative quantification in real-time RT-PCR. Nucleic Acids Res 29:e45. doi:10.1093/nar/29.9.e45 11328886 PMC55695

[44] Zianni M , Tessanne K , Merighi M , Laguna R , Tabita FR . 2006. Identification of the DNA bases of a DNAse I footprint by the use of dye primer sequencing on an automated capillary DNA analysis instrument. J Biomol Tech 17:103–113. 16741237 PMC2291779

[45] Kandler JL , Holley CL , Reimche JL , Dhulipala V , Balthazar JT , Muszyński A , Carlson RW , Shafer WM . 2016. The MisR response regulator is necessary for intrinsic cationic antimicrobial peptide and aminoglycoside resistance in Neisseria gonorrhoeae. Antimicrob Agents Chemother 60:4690–4700. doi:10.1128/AAC.00823-16 27216061 PMC4958169

[46] Ayala JC , Shafer WM . 2019. Transcriptional regulation of a gonococcal gene encoding a virulence factor (L-lactate permease). PLoS pathogens 15:e1008233. doi:10.1371/journal.ppat.1008233 31860664 PMC6957213

