# Supplementary material for: Transcriptional activation of ompA in Neisseria gonorrhoeae mediated by the XRE family member protein NceR
Source: mBio. 2023 Jun 30;14(4):e01244-23. doi: 10.1128/mbio.01244-23 (PMC10470515; doi:10.1128/mbio.01244-23)
Supplement: Supplemental material — Fig. S1 to S6 and Tables S1 to S4. [file mbio.01244-23-s0001.pdf]

Supplementary Information

Transcriptional activation of *ompA* in *Neisseria gonorrhoeae* mediated by the XRE family member protein NceR

Concerta L. Holley[1], Vijaya Dhulipala[1], Stavros A. Maurakis[2], Ashley Nicole Greenawalt[2], Timothy D. Read[3,4], Cynthia N. Cornelissen[2], and William M. Shafer[1,3,5*]

[1]Department of Microbiology and Immunology, [2]Institute for Biomedical Sciences, Georgia State University, Atlanta, GA, USA, [3]Department of Medicine (Division of Infectious Diseases) and [4]The Emory Antibiotic Resistance Center, Emory University School of Medicine, Atlanta, GA, USA, and [5]Laboratories of Bacterial Pathogenesis, Veterans Affairs Medical Center, Decatur, GA, USA

CONTENTS

Supplemental Figure S1. OmpA expression is increased in an iron-dependent, MisR-independent manner

Supplemental Figure S2. Fur represses *ngo1982* in the presence of Iron

Supplemental Figure S3. Alignment of the NceR sequence from different *Neisserial* strains using the ClustalW algorithm

Supplemental Figure S4. Genomic organization of the *N. gonorrhoeae ngo1982* locus

Supplemental Figure S5. Alignment of the NceR sequence from *N. gonorrhoeae* with selected XRE family member proteins from other bacterial strains using the ClustalW algorithm

Supplemental Figure S6. Predicted Structure of the NceR protein

Supplemental Table S1. Bacterial Strains and plasmids used in this study

Supplemental Table S2. DNA pull-down and Mass spectrometry identification of proteins interacting with the *ompA* promoter

Supplemental Table S3. Protein allele variants in the NceR found through analysis of sequenced clinical genomes

Supplemental Table S4. Primers Used in this study

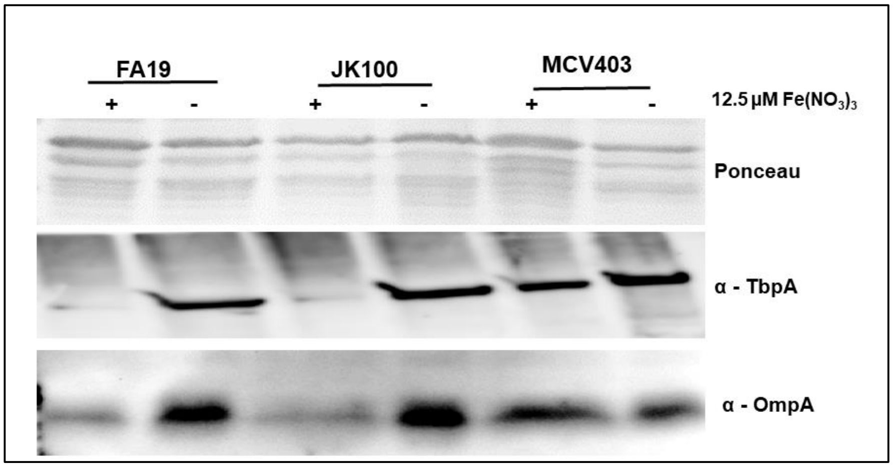

Figure S1. OmpA expression is increased in an iron-dependent, MisR-independent manner. Shown is a Western blot of 5 µgs of SDS-PAGE separated protein from whole cell lysates of isogenic Ng strains FA19, JK100, and MCV403 grown in the presence and absence of iron in CDM. The blot was probed with rabbit polyclonal anti-OmpA and anti-TbpA antiserum as described in Materials and Methods.

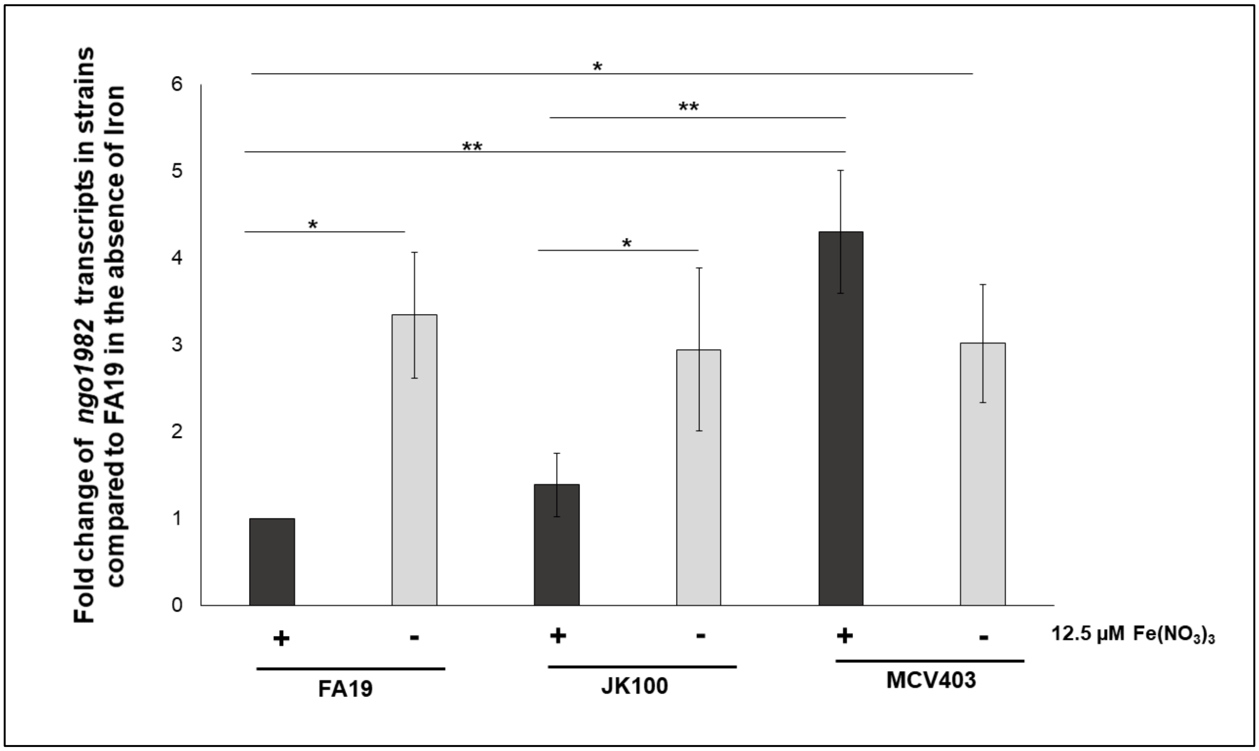

Figure S2. Fur represses *ngo1982* in the presence of Iron. qRT-PCR analysis of *ngo1982* transcripts in FA19, JK100, and MCV403 at the late-logarithmic phase of growth in the presence and absence of 12.5 µM $Fe(NO_3)_3$ in Chemically Defined Medium (CDM). Error bars represent standard deviations from the means of 3 independent experiments. Normalized expression ratios were calculated using *recA* rRNA expression and test values were further normalized against FA19 in the presence of $Fe(NO_3)_3$. The statistical significance of the results was determined by Student's t-test and adjusted for multiple comparisons, * = $P < 0.05$, ** = $P < 0.001$.

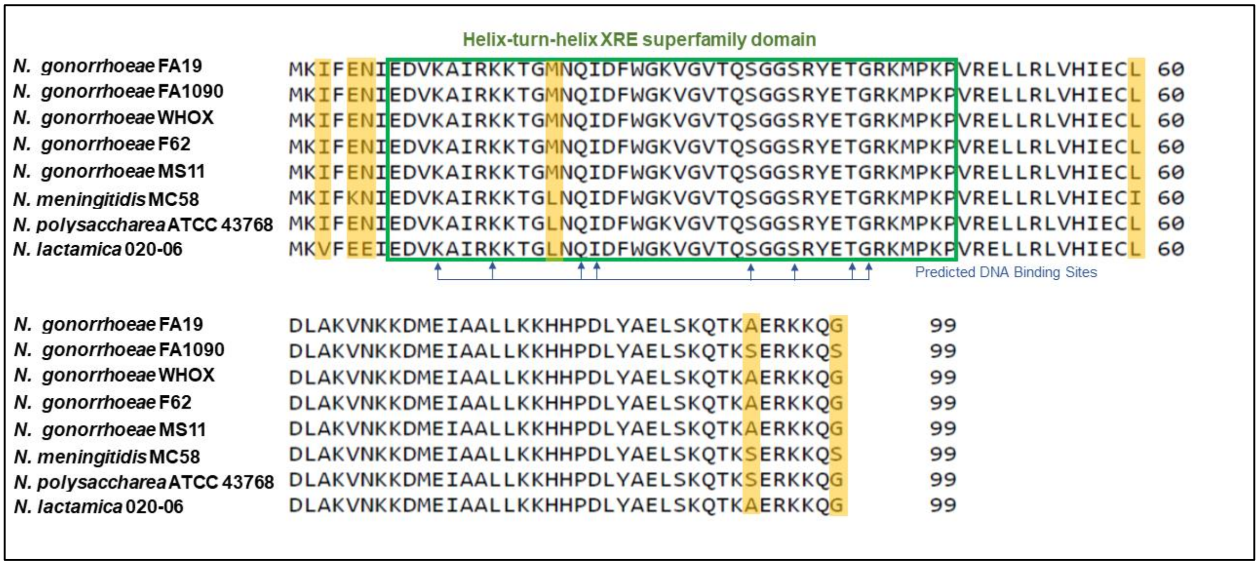

Figure S3. Alignment of the NceR sequence from different neisserial strains using the ClustalW algorithm. The helix-turn-helix XRE superfamily domain is boxed in green. Predicted DNA binding sites as identified by the NCBI Conserved Domain Database (1) are indicated with blue arrows. Amino acid differences between the strains are highlighted in gold. Locus IDs: *N. gonorrhoeae* FA19 (NGEG_RS0110135), *N. gonorrhoeae* FA1090 (F9Z35_RS05515), *N. gonorrhoeae* WHO X (C7S01_RS10830), *N. gonorrhoeae* F62 (NGNG_RS09285), *N. gonorrhoeae* MS11 (NGFG_RS11035), *N. meningitidis* MC58 (NMB_RS10940), *N. polysaccharea ATCC 43768* (NEIPOLOT_00329), and *N. lactamica 020-06* (NLA_RS01200).

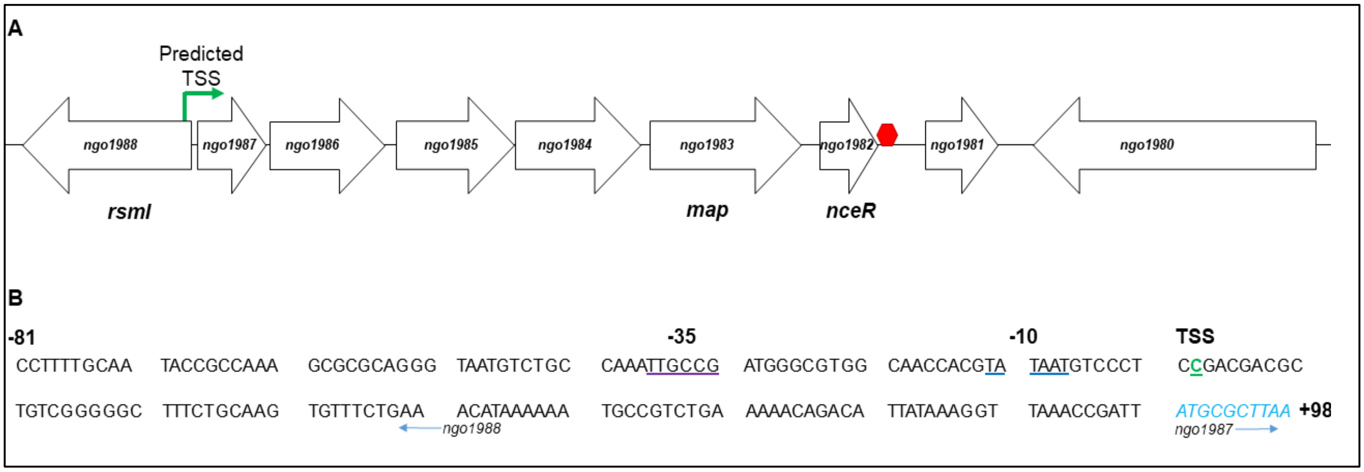

Figure S4. Genomic organization of the *N. gonorrhoeae ngo1982* locus. A) Relative position of the genes within the FA1090 strain genome (GenBank assembly accession number GCA_000006845.1). Approximate location of the predicted transcriptional start sites (TSS) of the operon based on transcriptome mapping of *N. gonorrhoeae* (2) is indicated with the green arrow. The approximate location of the transcript endpoint is indicated with a red hexagon. Map is drawn approximately to scale. B) Sequence of the NGO1987-NGO1982 operon promoter. The predicted -35, -10, and TSS are underlined.

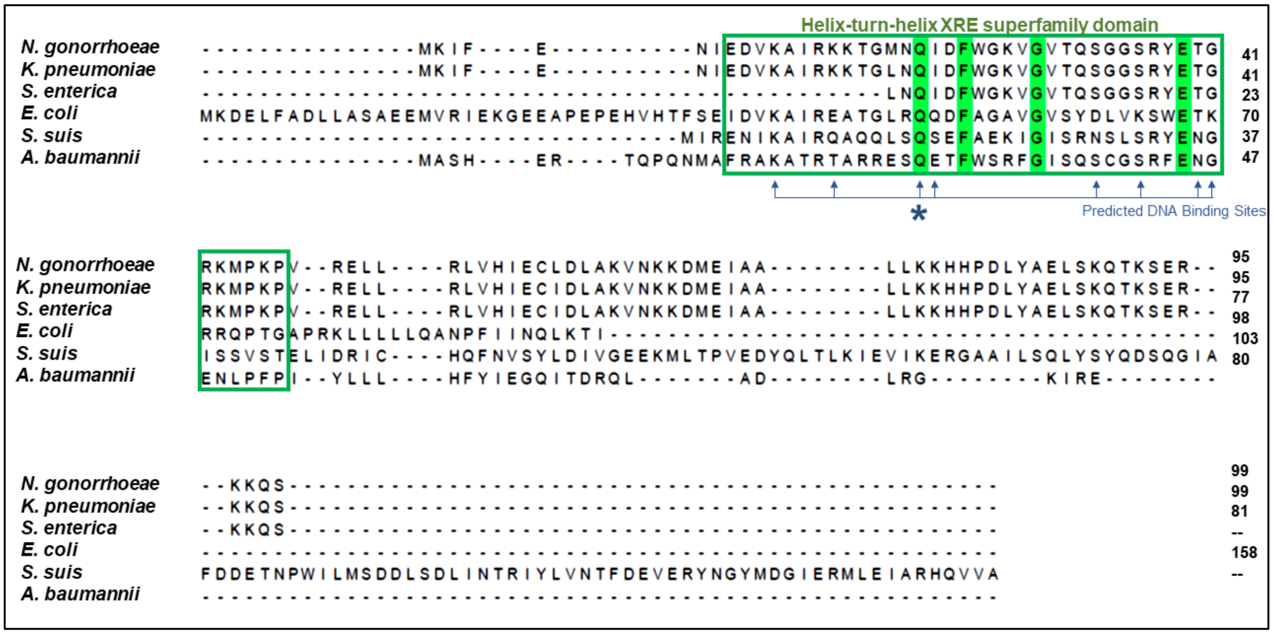

Figure S5. Alignment of the NceR sequence from *N. gonorrhoeae* with selected XRE family member proteins from other bacterial strains using the ClustalW algorithm. The helix-turn-helix XRE superfamily domain is boxed in green. Conserved residues between strains are highlighted in green. Predicted DNA binding sites as identified by the NCBI Conserved Domain Database (1) are indicated with blue arrows. A common predicted DNA binding site is denoted with an asterisk. Protein IDs: *N. gonorrhoeae* (WP_010951376.1), *K. pneumoniae* (MCI4052495.1), *S. enterica* (MBG8803505.1), *E. coli* (WP_170897976.1), *S. suis* (WP_024390985.1), and *A. baumannii* (SST11697.1).

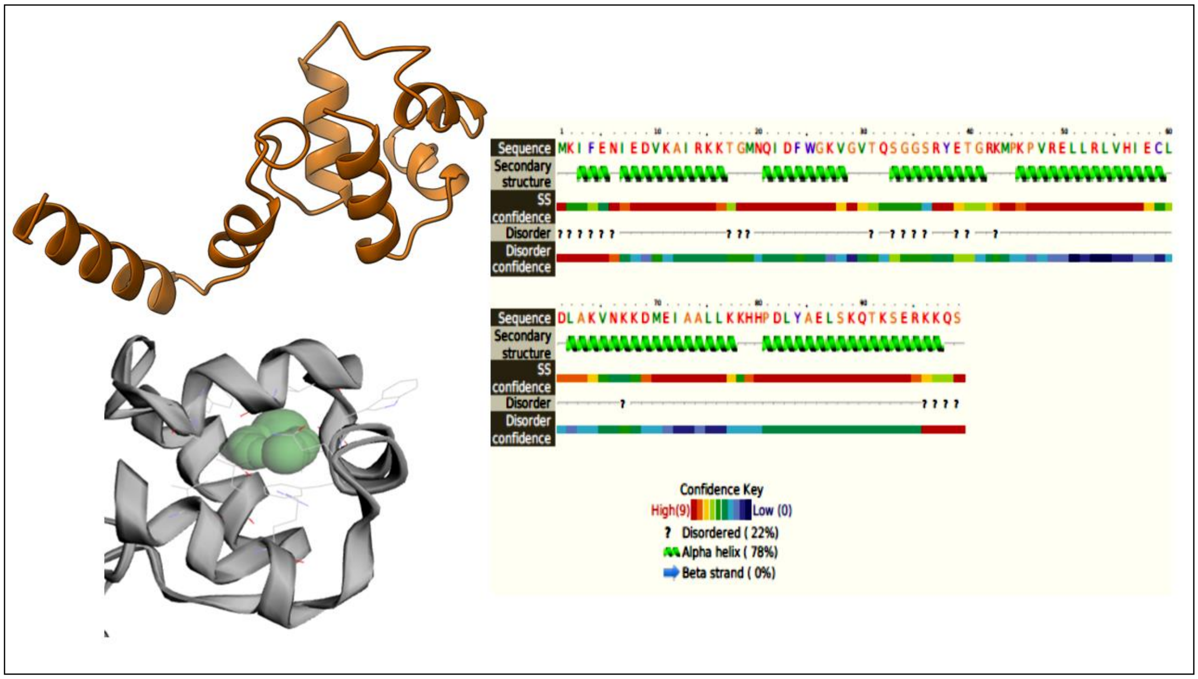

Figure S6. Predicted Structure of the NceR protein. Predicted structure of NceR is shown in yellow. A close-up of the predicted DNA binding pocket identified in the helix- turn- helix region of NceR is shown in green. Predicted secondary structures are shown above the amino acid sequence of NceR. Binding pocket prediction by CastP (3). Secondary structure prediction was performed using Phyre2 program (4).

Supplemental Table S1 - Bacterial Strains and plasmids used in this study

Strain or plasmid	Genotype or description	Reference or source
N. gonorrhoeae		
FA19 *rpsl*	WT strain	(5)
CH250	FA19 *rpsl* Δ*ngo1982*	This Study
CH258	FA19 *rpsl* Δ*ngo1982* pGCC4-*ngo1982*	This Study
JK100	FA19 *misR::kan*	(6)
MCV403	FA19 with the *fur-1* (Y82C) null allele	Gift from C. Cornelissen
CH10	FA19 *ompA::ermC*	(7)
FA19::P_{FL}	FA19 containing a translational fusion of 123 bp of the promoter region of *ompA* to the *lacZ* gene	(7)
FA19::$P_{FL\Delta S2}$	FA19 containing a translational fusion of 123 bp of the promoter region of *ompA* to the *lacZ* gene and S2 deletion	(7)
FA19::P_{Trunc}	FA19 containing a translational fusion of 81 bp of the promoter region of *ompA* to the *lacZ* gene	(7)
CH250::P_{FL}	CH250 containing a translational fusion of 123 bp of the promoter region of *ompA* to the *lacZ* gene	This study
CH300	CH250 containing a translational fusion of 123 bp of the promoter region of *ompA* to the *lacZ* gene and S2 deletion	This study
CH301	CH250 containing a translational fusion of 81 bp of the promoter region of *ompA* to the *lacZ* gene	This study
CH302	CH258 containing a translational fusion of 123 bp of the promoter region of *ompA* to the *lacZ* gene	This Study
CH303	CH258 containing a translational fusion of 123 bp of the promoter region of *ompA* to the *lacZ* gene and S2 deletion	This Study
Escherichia coli		
One Shot TOP10	F^- *mcr*A Δ(*mrr-hsdRMS-mcrBC*) ϕ80*lacZ*ΔM15 Δ*lacX74 recA1 araD139* (*ara leu*)*7697 galU galK rpsL* (Str^r) *endA1 nupG*	Invitrogen (Carlsbad, CA)
BL21(DE3)	*fhuA2 [lon] ompT gal (λ DE3) [dcm] ΔhsdS λ DE3 = λ sBamHIo ΔEcoRI-B int::(lacI::PlacUV5::T7 gene1) i21 Δnin5*	New England Biolabs (Ipswitch, MA)
**Plasmids**		
pET-15b	Bacterial expression vector with T7lac promoter, N-terminal His-tag	Merck Millipore (Burlington, MA)
pCH30	pET-15b containing FA19 *ngo1982* coding region	This Study
pUNCH937	pBluescriptII(SK-) derivative containing the sequential cloning of: *i*) a BamHI-EcoRI fragment encoding *rpsL* from pFLOB4300 (8) into BamHI-EcoRI and *ii*) The BglII *cat* cassette from pNC40 (9) into BamHI.	Christopher E. Thomas (University of North Carolina at Chapel Hill)
pCH31	pBad Δ*ngo1982*	This Study

pCH32	pbad Δ*ngo1982*::*rpls/CAT*	This Study
pGCC4	IPTG-inducible *Neisseria* chromosomal complementation vector	(10)
pCH33	pGCC4 containing the wild type *ngo1982* gene from FA19	This Study
pLES94	pUC18 derivative containing a truncated *lacZ* gene for use in translational fusions; recombines at the *proAB* locus of the gonococcal chromosome	(11)
pCH22	pLES94 containing 123 bp upstream of *ompA (FL)*	(7)
pCH23	pLES94 containing 81 bp upstream of *ompA (Trunc)*	(7)
pCH25	pCH22 with deleted S2 site	(7)

Supplemental Table S2. DNA pull-down and Mass spectrometry identification of proteins interacting with the *ompA* promoter

			FA19				JK100				
			+ iron		- iron		+ iron		- iron		Fold Change: -Fe /+Fe[a]
**Gene ID**	**Gene**	**Description**	Sample	Control (-DNA)	Sample	Control (-DNA)	Sample	Control (-DNA)	Sample	Control (-DNA)	
NGO_0177	*misR*	Two-component system transcriptional response regulator	**16**	0	**43**	0	0	0	0	0	--
NGO_0718	*ybbH*	Putative RpiR-family transcriptional regulator	**3**	0	**7**	0	**7**	0	**5**	0	2.06
NGO_0603	*ihfB*	Integration host factor subunit beta	**5**	0	**4**	0	**3**	0	**2**	0	--
NGO_2130	*regF*	RegF	**2**	0	**3**	0	**3**	0	0	0	2.3
NGO_1982		XRE family transcriptional regulator;Putative zinc finger/helix-turn-helix protein	**4**	0	**3**	0	**3**	0	**4**	0	9.95
NGO_1250	*mtrA*	HTH-type transcriptional regulator MtrA	0	0	**3**	0	0	0	0	0	--
NGO_0777	*dbhA*	Transcriptional regulator;DNA-binding protein Hu;Transcriptional regulator	0	0	**3**	0	0	0	**2**	0	2.27
NGO_0999	*rpoD*	RNA polymerase sigma factor RpoD	0	0	**3**	0	0	0	**3**	0	2.77
NGO_0305	*ihfA*	Integration host factor subunit alpha	**2**	0	**2**	0	**2**	0	**2**	0	2.36
NGO_0322		Uncharacterized protein	0	0	**2**	0	0	0	0	0	78.8
NGO_2122		Uncharacterized protein	0	0	**2**	0	0	0	0	0	--
NGO_1043		Uncharacterized protein	0	0	0	0	0	0	**2**	0	3.8
NGO_1613		Putative integrase/recombinase phage associated protein	0	0	0	0	0	0	**3**	0	--

[a]Iron and/or Fur regulated as described in (12)

Supplemental Table S3 : Protein allele variants in NceR found through analysis of sequenced clinical genomes[a]

Protein allele group	AA changes[b]	Total # of Isolates in Group	% of Strains in dataset with allele variant
WP_003686868.1	Wild-Type	626	81.6%
WP_002215072.1	M19L, L60I, A93S, G99S	115	15.0%
WP_002243124.1	M19L, L60I , G99S	7	0.91%
WP_172760178.1	G35D	5	0.65%
WP_172763779.1	[deletion AA 1-10]	2	0.26%
WP_010951376.1	A93S, G99S	1	0.13%
WP_048339600.1	S36P	1	0.13%
WP_071197876.1	[deletion AA 1-18]	1	0.13%
WP_139594787.1	S36L	1	0.13%
WP_172763623.1	N6I, G35D	1	0.13%
WP_172765896.1	[deletion AA 1-6]	1	0.13%
WP_172766211.1	[deletion AA 1-7], G34R	1	0.13%
WP_192213791.1	G41C, R42S, A93S	1	0.13%
WP_192214471.1	A93S	1	0.13%
WP_192276056.1	G41C, A93S	1	0.13%
WP_192370388.1	G41D, A93S	1	0.13%
WP_192390701.1	G41Y, A93S	1	0.13%

[a] = Comparison of 767 public genome projects for *N. gonorrhoeae* strains

[b] = Compared to FA1090 (Reference Number: WP_010951376.1)

Supplemental Table S4 – Primers Used in this study

Primer Name	Sequence (5' to 3') †	Purpose
ompA_PD_F	[BTN]TCGGTTCCGTACTATCTGTACTGT	DNA pull-down
ompA_PD_R	GGTTTGAAGAAAGTCATGGCG	DNA pull-down
1982F1	TGCGGTGGTCAGAAGTTTCA	Construction of *ngo1982* mutant; sequencing primer
1982R1	AACCGAAACCGGCTACGAAA	Construction of *ngo1982* mutant; sequencing primer
1982start	TCTAGAAAGCTTAAATATTTTCATAAAACT	Construction of *ngo1982* mutant
1982stop	TCTAGACAAAGTTAAACCGCGACC	Construction of *ngo1982* mutant
1982pacI	ATTAATTAAGTCAGCCCCGCCACC	Complementation of *ngo1982* mutant
1982pmeI	GGGTTTAAACCGTTTCGGACTGTTTG	Complementation of *ngo1982* mutant
1982HisF	TATACATATGATGAAAATATTTGAAAAT	Construction of NceR-His plasmid
1982HisR	TATAGGATCCTTAACTTTGTTTTTTTCTTTCG	Construction of NceR-His plasmid
recAqFw	AACCTCGAAGTCATTTCCACCGG	qRT-PCR
recAqRv	TCTGGCATTGGGCGACGGCTTC	qRT-PCR
1982 qRTF	AAGATGTTAAAGCCATCCGTAAA	qRT-PCR
1982 qRTR	ATTGAGTAACGCCGACCTT	qRT-PCR
ompA_qRTF	CCCAAACAAATCCGCCATGT	qRT-PCR
ompA_qRTR	CGCTGCTCTTGGTAGTCCATAT	qRT-PCR
1981_RT_F	CAATCCTGTCTTCCGCAATC	qRT-PCR
1981_RT_R	TCAGCACATAACCGCCTTC	qRT-PCR
mtrR qRT F	CTTGTTTGACGCGTTGTTCCA	qRT-PCR
mtrR qRT R	GTGGATGTCGTTGCTTTGCA	qRT-PCR
ltgA qRT F	AAGAAGCACGCAAATCGCC	qRT-PCR
ltgA qRT F	TGATAATGCCCCAATACGCC	qRT-PCR
pOmpA2F	CCGCCTTAGCTCAAAGAGAA	EMSA
pOmpAR	TTAAGAATTCCGCCACCCAAACCGTACAT	EMSA
[FAM]ompAFL	[6-FAM] AGTGAATCGGTTCCGTACTATCTGTACTGT	DNAse I protection
[Hex]ompR	[HEX]GAAAGTCATGGCGTTTCCTT	DNAse I protection
ompAFL	AGTGAATCGGTTCCGTACTATCTGTACTGT	Sequencing Ladder
ompR	GAAAGTCATGGCGTTTCCTT	Sequencing Ladder
lctp	GCGCGATCGGTGCGTTCG	Sequencing primer
aspC1	GCCGGATGCGTCTTTGTAC	Sequencing primer

† Restriction endonuclease cut sites are underlined. BTN= biotin

Supplemental References

1. Lu S, Wang J, Chitsaz F, Derbyshire MK, Geer RC, Gonzales NR, Gwadz M, Hurwitz DI, Marchler GH, Song JS, Thanki N, Yamashita RA, Yang M, Zhang D, Zheng C, Lanczycki CJ, Marchler-Bauer A. 2020. CDD/SPARCLE: the conserved domain database in 2020. Nucleic Acids Res 48:D265-D268.
2. Remmele CW, Xian Y, Albrecht M, Faulstich M, Fraunholz M, Heinrichs E, Dittrich MT, Muller T, Reinhardt R, Rudel T. 2014. Transcriptional landscape and essential genes of *Neisseria gonorrhoeae*. Nucleic Acids Res 42:10579-95.
3. Tian W, Chen C, Lei X, Zhao J, Liang J. 2018. CASTp 3.0: computed atlas of surface topography of proteins. Nucleic Acids Res 46:W363-W367.
4. Kelley LA, Mezulis S, Yates CM, Wass MN, Sternberg MJ. 2015. The Phyre2 web portal for protein modeling, prediction and analysis. Nat Protoc 10:845-58.
5. Sarubbi FA, Jr., Blackman E, Sparling PF. 1974. Genetic mapping of linked antibiotic resistance loci in *Neisseria gonorrhoeae*. J Bacteriol 120:1284-92.
6. Kandler JL, Holley CL, Reimche JL, Dhulipala V, Balthazar JT, Muszynski A, Carlson RW, Shafer WM. 2016. The MisR Response Regulator Is Necessary for Intrinsic Cationic Antimicrobial Peptide and Aminoglycoside Resistance in *Neisseria gonorrhoeae*. Antimicrob Agents Chemother 60:4690-700.
7. Holley CL, Ayala JC, Shafer WM. 2020. Transcriptional control of the gonococcal ompA gene by the MisR/MisS two-component regulatory system. Sci Rep 10:9425.
8. Johnston DM, Cannon JG. 1999. Construction of mutant strains of *Neisseria gonorrhoeae* lacking new antibiotic resistance markers using a two gene cassette with positive and negative selection. Gene 236:179-84.
9. Thomas CE, Carbonetti NH, Sparling PF. 1996. Pseudo-transposition of a Tn5 derivative in *Neisseria gonorrhoeae*. FEMS Microbiol Lett 145:371-6.

10. Mehr IJ, Seifert HS. 1998. Differential roles of homologous recombination pathways in *Neisseria gonorrhoeae* pilin antigenic variation, DNA transformation and DNA repair. Mol Microbiol 30:697-710.

11. Silver LE, Clark VL. 1995. Construction of a translational lacZ fusion system to study gene regulation in *Neisseria gonorrhoeae*. Gene 166:101-4.

12. Ducey TF, Carson MB, Orvis J, Stintzi AP, Dyer DW. 2005. Identification of the iron-responsive genes of *Neisseria gonorrhoeae* by microarray analysis in defined medium. J Bacteriol 187:4865-74.
